# Emergency department clinicians’ views on implementing psychosocial care following acute paediatric injury: a qualitative study

**DOI:** 10.1080/20008066.2023.2300586

**Published:** 2024-01-10

**Authors:** Nimrah Afzal, Mark D. Lyttle, Mohsen Rajabi, Frankie Rushton-Smith, Rhea Varghese, David Trickey, Sarah L. Halligan, on behalf of the Paediatric Emergency Research in the UK and Ireland (PERUKI)

**Affiliations:** a Department of Psychology, University of Bath, Bath, UK; b Emergency Department, Bristol Royal Hospital for Children, Bristol, UK; c Research in Emergency Care Avon Collaborative Hub (REACH), University of the West of England, Bristol, UK; d Anna Freud Centre, UK Trauma Council, London, UK

**Keywords:** PTSD, trauma, emergency care, paediatric trauma, trauma-informed care, TEPT (Trastorno de estrés postraumático), trauma, atención de urgencia, trauma pediátrico, servicios informados en trauma

## Abstract

**Introduction:** The early post-trauma period is a key time to provide psychological support to acutely injured children. This is often when they present to emergency departments (EDs) with their families. However, there is limited understanding of the feasibility of implementing psychological support for children and their families in EDs. The aim of this study was to explore UK and Irish ED clinicians’ perspectives on developing and implementing psychosocial care which educates families on their children’s post-trauma psychological recovery.

**Methods:** Semi-structured individual and group interviews were conducted with 24 UK and Irish ED clinicians recruited via a paediatric emergency research network.

**Results:** Clinicians expressed that there is value in offering psychological support for injured children and their families; however, there are barriers which can prevent this from being effectively implemented. Namely, the prioritisation of physical health, time constraints, understaffing, and a lack of training. Therefore, a potential intervention would need to be brief and accessible, and all staff should be empowered to deliver it to all families.

**Conclusion:** Overall, participants’ views are consistent with trauma-informed approaches where a psychosocial intervention should be able to be implemented into the existing ED system and culture. These findings can inform implementation strategies and intervention development to facilitate the development and delivery of an accessible digital intervention for acutely injured children and their families.

## HIGHLIGHTS

- The emergency department provides an opportunity for early trauma-informed care for acutely injured children and their families.
- Addressing psychological distress in emergency care for acutely injured children and their families should adopt a universal trauma-informed approach.
- The development of a paediatric trauma-informed intervention should consider barriers which can impact implementation into emergency care. Particular barriers highlighted by clinicians include staff shortages, time constraints, and high caseloads.

## Abstract (es)

**Introducción:** El periodo postraumático temprano es un momento clave para proporcionar apoyo psicológico a los niños con lesiones agudas. A menudo, esto ocurre cuando acuden a los servicios de urgencias (SU) con sus familias. Sin embargo, existe una comprensión limitada sobre la viabilidad de implementar apoyo psicológico para niños y sus familias en los SU. Sin embargo, existe una limitada comprensión sobre la viabilidad de la implementación de apoyo psicológico para niños y sus familias en SU. El objetivo de este estudio fue explorar las perspectivas de los médicos de urgencias del Reino Unido e Irlanda sobre el desarrollo y la aplicación de la atención psicosocial que educa a las familias en la recuperación psicológica de sus hijos después del trauma.

**Método:** Se realizaron entrevistas semiestructuradas individuales y en grupo a 24 médicos del SU del Reino Unido e Irlanda reclutados a través de una red de investigación en urgencias pediátricas.

**Resultados:** Los médicos expresaron que ofrecer apoyo psicológico a los niños heridos y a sus familias tiene valor; sin embargo, existen obstáculos que pueden dificultar que esto se lleve a cabo de forma eficaz. Específicamente, la priorización de la salud física, las limitaciones de tiempo, la falta de personal y la falta de formación. Por lo tanto, una posible intervención tendría que ser breve y accesible, y todo el personal debería estar capacitado para ofrecerla a todas las familias.

**Conclusiones:** En general, las opiniones de los participantes son coherentes con los enfoques informados en trauma, donde una intervención psicosocial debería poder implementarse en el sistema y la cultura existentes del SU. Estos hallazgos pueden dar información sobre la intervención y la implementación de estrategias para facilitar el desarrollo y entrega de intervenciones digitales accesibles para niños con trauma agudo y sus familias.

## 1. Introduction

Between 2 and 3 million children and adolescents aged 0-17 years old have attended UK emergency departments (EDs) between 2020 and 2021 (NHS Digital, 2021), with accidental injuries being common reasons for admissions (e.g. motor vehicle accidents, burns) (Public Health England, 2018). Accidental injuries in children can cause post-traumatic stress symptoms (PTSS) (Landolt et al., 2003), including hyperarousal, re-experiencing the traumatic event, intrusive thoughts relating to the traumatic experience, and cognitive and emotional changes (American Psychiatric Association, 2013). Estimates suggest that up to 16% of trauma-exposed children will develop persistent post-traumatic stress disorder (PTSD) following acute trauma (e.g. disasters, acute injury, violence-related injuries) (Hiller et al., 2016; Ophuis et al., 2018; van Meijel et al., 2019). Other longer-term consequences include substance abuse, depression, anxiety and functional impairment into adolescence and adulthood (Downey & Crummy, 2022; Dye, 2018; Holbrook et al., 2005). However, despite the significant disability associated with PTSD, only a minority of trauma-exposed children seek help for PTSS, and even fewer have received support from mental health professionals (Lewis et al., 2019). Therefore, it is important to consider how current healthcare practices can be improved to provide timely and appropriate support to trauma-exposed children (Kazak et al., 2006).

Early interventions provide an opportunity to alleviate acute distress and prevent chronic PTSD development (Zatzick et al., 2009). Critically, hospital EDs present a unique opportunity for providing early intervention in the hours immediately following a traumatic event (Kearns et al., 2012). Research suggests that integrating mental and physical healthcare within emergency medical settings has the potential to improve outcomes of psychological distress, as well as improving medical treatment adherence (Horowitz et al., 2001; Marsac et al., 2018). Models of child PTSS following paediatric injury conceptualise how medical teams can provide psychological and emotional support. For example, the Paediatric Medical Traumatic Stress model (Kazak et al., 2006; Price et al., 2016) and the bio-psycho-social model of PTSS (Marsac et al., 2014) each highlight the value of clinicians providing anticipatory guidance about emotional recovery to children and families, and screening for potential PTSS. Therefore, the early-post trauma period provides an opportunity for clinicians to provide low-intensity and timely support for children’s post-trauma psychological recovery.

Research has provided promising results in relation to the feasibility and/or effectiveness of providing anticipatory guidance to children and their families following ED attendance. For example, the Coping with Accident Reactions intervention (De Young et al., 2016; Haag et al., 2020), a psychologist-delivered psychoeducation for parents of unintentionally injured children, has been found to reduce child PTSS severity within the first three months post-injury. Other parent-focused psychoeducational interventions have similarly shown promising results in terms of acceptability for parents (Marsac et al., 2018, 2019), improvements in parents’ functional and emotional coping (Melnyk et al., 1997, 2004), and in decreasing children’s anxiety (Cox et al., 2010; Kenardy et al., 2010) in paediatric emergency populations. These findings suggest that there is a potential benefit to providing anticipatory guidance for children and their families following hospitalisation*.* However, the evidence-base is still emerging, and findings are limited to the United States and Australia. Moreover, the above interventions are delivered directly to parents and the feasibility of delivering such interventions via ED teams is still under-explored.

Qualitative work conducted in the UK with parents of trauma-exposed children reflects parents’ need for guidance about their child’s psychological recovery from healthcare professionals, including in ED settings (Williamson et al., 2016; Wiseman et al., 2018). However, a detailed understanding of what support ED staff could potentially provide is currently lacking. Findings from a worldwide survey identified that ED clinicians required greater training to improve their knowledge of and confidence in providing support for paediatric traumatic stress (Alisic et al., 2016). National subsets of this data have been analysed, with analyses indicating that ED staff report little to no training in paediatric traumatic stress and low rates of confidence in educating parents and injured children about common traumatic stress symptoms in Australia and New Zealand (Hoysted et al., 2017) and in the UK and Ireland (Afzal et al., 2022). In the UK and Ireland, ED staff highlighted specific barriers to providing psychosocial care, including time constraints, a lack of relevant training and a confusing evidence-base (Afzal et al., 2022). Altogether, these findings indicate that there are specific aspects of the ED environment and culture which may influence the feasible and sustainable implementation of psychosocial care into EDs.

The present study aimed to build on the above-described quantitative evidence of barriers to providing psychosocial care among ED staff, by conducting an in-depth, qualitative investigation into UK and Irish ED clinicians’ perspectives on providing psychosocial care to injured children and families. There has been limited qualitative examination so far, particularly within the UK and Ireland. Implementation research highlights the importance of qualitative research for an in-depth exploration of the environment in which potential clinical innovations will be implemented (Hamilton & Finley, 2019). As such, we included a detailed examination of key practical considerations relating to intervention delivery, in order to inform future development of evidence-based implementation strategies suitable for EDs. Thus, the present study sought to explore ED clinicians’ views on the feasibility, acceptability, appropriateness, and sustainability of delivering psychosocial care (Proctor et al., 2011).

The specific aims of this qualitative study were to investigate ED clinicians’ views on: (1) whether it is feasible to deliver a psychosocial intervention to children and families in the ED, (2) who is best placed to deliver such care, and (3) how to deliver a potential intervention feasibly and appropriately in light of ED pressures. These findings can inform an understanding of what future research must consider in adapting existing interventions in the UK and Ireland.

## 2. Methods

We conducted a qualitative study with paediatric emergency research clinicians within the UK and Ireland. The University of Bath Psychology Research Ethics Committee approved the study (PREC: 22-021).

### 2.1. Participants

Participants were recruited through advertising via the paediatric emergency research network in the UK and Ireland (PERUKI: Lyttle et al., 2014) and subsequently via snowballing. PERUKI is a multicentre research network consisting of 73 sites across England, Ireland, Northern Ireland, Scotland, and Wales, spanning paediatric-specific and mixed adult/paediatric EDs.

Participants were eligible if they were currently working as an ED clinician (doctors, nurses, practitioners, psychologists, and psychiatrists). Participants registered for the study via an online form which collected basic professional characteristics (e.g. role, level of expertise, years of experience in the ED, and clinical environment). Participants provided informed consent prior to taking part in an individual interview or a small group interview. Participants were given a £25 voucher following their participation.

Forty-five clinicians volunteered to take part in the study, and 33 were invited to take part in either a group or individual interview. Nine of the 33 responded ‘yes’ but were subsequently not contactable, resulting in a final sample of 24 participants. Further participants were not approached as by this point, data saturation had been achieved (Hennink & Kaiser, 2022).

### 2.2. Interviews

A semi-structured interview schedule was developed (see Table 1), guided by previous research exploring ED clinicians’ perspectives on psychological care for injured children, which highlighted key barriers including time constraints, a lack of knowledge in paediatric PTS and low confidence in aspects of psychosocial care (Afzal et al., 2022; Alisic et al., 2016). The interview was framed to explore clinicians’ views on the implementation of psychosocial support in the form of a psychoeducational intervention for children and families following acute paediatric injury, with close consideration of these previously identified barriers. The interview schedule was designed to assess specific aspects of intervention implementation: feasibility (i.e. the extent to which a potential intervention/source of support can be practically and realistically delivered in the ED), acceptability (i.e. how to ensure that intervention content, complexity, and delivery is acceptable for the clinicians and patients), appropriateness (i.e. whether such care is appropriate to provide in the ED), and sustainability (i.e. how can support can be maintained over time) (Hamilton & Finley, 2019; Proctor et al., 2011).

**Table 1.** Interview topic guide with questions and follow-up questions.

Is the ED the right place to offer mental health support/implement a psychosocial intervention for acutely injured children? What is the ideal mode of delivery to implement a potential psychoeducational intervention into the ED? Which families should receive psychosocial support/a potential intervention? *Will clinicians be able to recognise those who require further support? What would this involve?* Who is best placed to provide psychosocial support or deliver a potential intervention? *Should this be the responsibility of clinicians from a single profession (e.g. nurses or doctors)?* At what point in the ED visit should the support/intervention be offered? *How would this fit into the child’s medical care?* Would a training package be useful, and, if so, what should this include? *Who should receive training?* How confident are you to provide a potential intervention to families? *What is required to increase your confidence?*

Selection into either group or individual interviews was not based on participant characteristics. Participants were offered group interviews as a first option, as we considered that these could be beneficial in facilitating discussion between clinicians with different roles from different hospital trusts. However, given the severe constraints imposed by clinical work patterns, we also offered one-to-one interviews with the primary researcher (N.A.). All interviews took place online (M.S. Teams).

Participants were provided with an information sheet prior to the interviews, which outlined key details regarding the aims of the study, a brief interview guide, and the importance of providing children and families with psychosocial support following acute paediatric injury. Prior to initiating the interviews, participants were briefed on the kind of intervention the investigators were seeking to explore the implementation of (i.e. a psychoeducational resource providing children and families with anticipatory guidance regarding paediatric PTS recovery), with previous interventions used as examples (e.g. De Young et al., 2016; Marsac et al., 2018).

Participants were informed of key findings following data analysis.

### 2.3. Data analysis

Interviews were recorded and transcribed verbatim. The primary researcher (N.A.) analysed the resulting data using NVivo, following Braun & Clarke's (2006, 2021) six-step approach to inductive thematic analysis. This approach was used as it is a ‘data-driven’ approach where codes and themes solely reflect participants’ views, free from pre-conceived theories and coding frameworks. The six steps are: (1) read and re-read transcripts to ensure familiarisation with the data, (2) generate initial codes, (3) generate themes and sub-themes, (4) review potential themes, (5) define and name themes, and (6) produce the report. Data from group and individual interviews were analysed concurrently, using the same methodology of inductive thematic analysis, which is consistent with existing research (e.g. Grant et al., 2016). This method of analysis was used because the same interview schedule was utilised for both individual and group interviews, and there was no evidence that findings differed during analyses.

First, all transcripts were read by N.A. to produce an initial list of codes. Following this, the transcripts were re-read to review codes and ensure that important information was not missed. Close attention was given to participants’ views on barriers and facilitators to implementation, and views on providing mental health support in the ED. The initial codes were then collated into a list to identify overarching themes.

Further steps to ensure integrity and coding trustworthiness were taken by including a second coder. Second coding is an important means of establishing trustworthiness and ensuring confidence for research with real-world applications and clinical recommendations (O’Connor & Joffe, 2020). Nowell et al. (2017) provide means of establishing trustworthiness in thematic analysis, including researcher triangulation which includes vetting themes and subthemes with team members and achieving a team consensus.

Therefore, a second coder (M.R.) independently read 20% of the transcripts (2 group interviews and 2 individual interviews) and produced a list of initial codes and themes. Authors (N.A. and M.R.) met to compare codes and themes, and, overall, similar codes and themes emerged from each analysis. Discussion facilitated a revision of the thematic structure, with a key focus on the authors working together to define themes clearly. Following this, the first author (N.A.) reviewed the final themes against all of the coded interview segments. These final themes were vetted through peer debriefing.

## 3. Results

The final sample consisted of 12 nurses (including two nurse practitioners), 10 doctors, and two others (physiotherapists and psychologists). On average, participants had worked in the ED for 8.00 years (range: 2–20 years, *SD* = 5.65). Full demographic characteristics of the final sample are described in Table 2.

**Table 2.** Descriptive statistics.

Characteristic	Statistic
Sex, *n* (%)	
Female	16 (66.7%)
Male	8 (33.3%)
Profession, *n* (%)	
Doctor	10 (41.7%)
Nurse (including nurse practitioners)	12 (50.0%)
Other	2 (8.3%)
Years of ED experience, *M(SD)*	8.00 (5.65)
Country, *n* (%)	
England	19 (79.2%)
Ireland	1 (4.2%)
Scotland	1 (4.2%)
Wales	3 (12.5%)
Stage of Care, *n* (%)	
Acute Phase Only	16 (66.7%)
Inpatient Care/Rehab Only	3 (12.5%)
Both	5 (20.8%)
Department, *n* (%)	
Paediatric ED	16 (66.7%)
Combined paediatric and adult ED	6 (25.0%)
Inpatient	1 (4.2%)
Trauma Coordinator	1 (4.2%)
Hospital Category, *n* (%)	
Paediatric major trauma centre	10 (41.7%)
Combined paediatric/adult major trauma centre/unit	12 (50.0%)
None of the above	2 (8.3%)

Four group interviews and 11 individual interviews were conducted. A summary of participants and whether they took part in a group or individual interview is provided in Table 3.

**Table 3.** Interview participation summary.

Participant identification number	Occupation	Grade	Years of experience	Group/individual interview	Group interview number
1	Doctor	Consultant	20	Individual	N/A
2	Doctor	Consultant	12.5	Individual	N/A
3	Nurse	Band 6	3	Individual	N/A
4	Doctor	Specialty trainee years 4-8	9	Group	1
5	Physiotherapist/AHP	N/A	N/A	Group	1
6	Nurse	Band 7	7	Individual	N/A
7	Nurse practitioner	Band 7	20	Individual	N/A
8	Doctor	Consultant	3	Individual	N/A
9	Doctor	Consultant	10	Individual	N/A
10	Nurse	Band 5	2	Individual	N/A
11	Nurse	Band 5	1.5	Individual	N/A
12	Nurse practitioner	Band 8	10	Group	2
13	Nurse	Band 7	7	Group	2
14	Nurse	Band 6	7	Group	2
15	Psychologist	Staff Grade or Associate Specialist	3	Group	2
16	Nurse	Band 7	N/A	Individual	N/A
17	Doctor	Consultant	15	Group	3
18	Doctor	Consultant	18	Group	3
19	Doctor	Consultant	4	Group	3
20	Nurse	Band 7	7	Group	3
21	Nurse	Band 6	5	Group	3
22	Doctor	Consultant	PEM Grid, 2 years post CCT	Group	4
23	Doctor	Consultant	17	Group	4
24	Nurse	Band 5	4	Group	4

### 3.1. Thematic analysis findings

**Figure 1.**
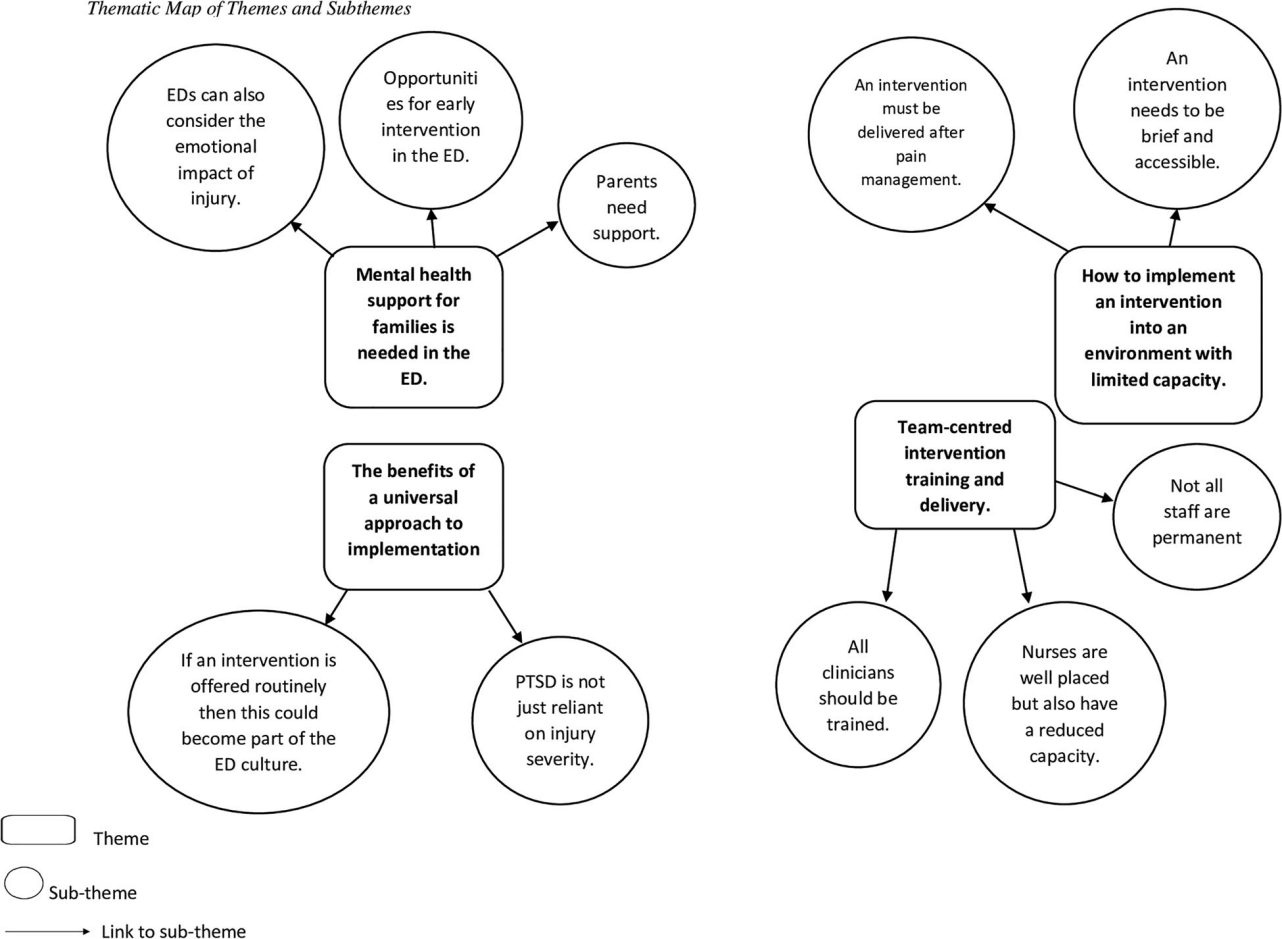
Final thematic map of themes and sub-themes.

Four overarching themes emerged from the data which captured clinicians’ views on the value of offering families mental health support, as well as the barriers and facilitators to implementing an intervention supporting post-injury mental wellbeing. A thematic map of the final themes and sub-themes is presented in Figure 1. Supplementary Material A provides the list of themes and sub-themes with multiple sample quotations.

#### 3.1.1. Theme One: mental health support for families is needed in the ED

With regard to the appropriateness of providing mental health support in the ED, clinicians saw value in offering a potential intervention which supports the psychological wellbeing of families. All clinicians expressed an understanding that injuries can have a psychological impact upon children and families. Some clinicians highlighted that the ED provides a unique opportunity for providing support (e.g. anticipatory information) in the early post-trauma period and that (medical) care could also include mental health.

##### 3.1.1.1. Subtheme One: EDs can also consider the emotional impact of injury

A number of clinicians understood that an injury can also result in psychological, as well as physical, consequences for children and families. A minority also discussed the value of providing information about mental healthcare, as well as physical healthcare, extending current practices beyond just physical care.

We're giving anaesthesia, we’re monitoring patients, but we’re monitoring multiple patients and you know managing them all as best we can. So, in the times that we are not at the patient’s side, giving parents something that is going to educate them on the impact of trauma and what they can do to better understand those impacts and how they can, um, not change their behaviour, but how they can manage that as best as possible and I think it's really, really valuable. (PID 003, Nurse)

Clinicians noted the need to prioritise acute medical needs that arise following trauma versus the potential longer term, psychological impact on children and families. However, the consequent limited attention to psychological health when caring for families was noted as a possible limitation.

I think it is an area where we probably fail to and fully meet children and families’ needs. I think we see children and they've been involved in quite high mechanism accidents. And we often do their scan and their scan comes back negative and we feel quite relieved that we can kind of turn them around and get them home often and probably don't think a huge amount about what the aftermath from a psychological perspective is and yeah, I think it probably is more important than we give it credit, really. (PID 009, Doctor)

##### 3.1.1.2. Subtheme Two: opportunities for early intervention in the ED

Clinicians highlighted their contact with families and children during a key, vulnerable period on presentation to the ED. This was seen as important because it can provide the opportunity to get families’ attention early on, which may be difficult once they are outside of this acute healthcare setting.

I think it's having that early opportunity to, to intervene and support at that (.) umm, point that that young person is either, you know, at their most vulnerable or with the most questions, and the most in crisis, most in need of that support. And so, you jumping in at that very early opportunity is gonna make all the difference, I think, to being able to support that child, and their family going forward. (PID 023, Doctor)

The acute care context was also viewed as a potential challenge as families may be too overwhelmed to engage with psychological support. Therefore, some clinicians emphasised that whilst the ED was a good place to initiate support, the amount of information provided to families must be considered carefully.

And so there’s a place for kind of going, ‘You know there’s this thing out here and here’s some signposting to it,’ but recognizing that maybe they’re not going to engage with it right now because they’re too busy processing the fact that this isn’t the way they expected their day to turn out. (PID 018, Doctor)

##### 3.1.1.3. Subtheme Three: parents need support

Clinicians shared experiences of interactions with parents and highlighted that the child’s traumatic event can also be distressing for parents and that it is often parents who need emotional support. Parental symptoms, such as flashbacks, emotional distress, and guilt around the child’s traumatic experience, were highlighted.

And sometimes I think also the scenario or the mechanism that led to the injury, I've seen a lot of times and if parents have had some sort of a contributing factor or even if they haven't, they often tend to blame themselves for not being around or for, you know, anything that they could have done preventative to have you avoided them from being in that situation. In those situations I think it's kind of important to be able to give even parents some support. It's not just a child, I think. Parents do have a lot of impact, uh themselves. (PID 004, Doctor)

Empowering parents in supporting their child was also seen as a route to improving family wellbeing overall.

But what I think I do recognize is that parents, particularly when they've something significant has happened, can feel quite helpless. … . When you give parents quite robust information, I think they go home feeling more comfortable and I imagine reducing parental anxiety is likely to have a ripple effect throughout the household and also to the child. So I imagine there's a probably a huge benefit and the kind of family attitude towards the event I imagine. (PID 009, Doctor)

#### 3.1.2. Theme Two: the benefits of a universal approach to implementation

When considering the appropriateness and feasibility of providing psychosocial care, the majority of clinicians expressed a benefit in offering an intervention for all families. With regard to appropriateness, many clinicians expressed that it would be fair to offer an intervention to all families of injured children. This was viewed as important because the ED provides an opportunity for early intervention, which may be less accessible for families once they leave the hospital. Therefore, providing support to all families of injured children would be a fair way to reach the target population.

But sometimes it's just not right for that particular young person or child at that time, and as long as you can identify that and say OK here are the resources, you don't need to access them now, don't worry about it, but take this away with you (.) so at some point, if you want to, then you can access these resources. Because by that time, the (.) the face-to-face contact with me or the other clinician or, or whoever it is has been lost. You're not gonna get that a year later or six months later, but at least you've got the resources. (PID 024, Nurse)

In terms of feasibility, some clinicians emphasised that the business of the ED means that it can be difficult to identify who may or may not need the intervention. Therefore, a blanket approach to offering the intervention could help overcome barriers including time-constraints and caseloads.

It's difficult really because stress will kind of affect people so differently. It's kind of who do you target to give the advice to. [The] ED is ridiculously busy. I think we saw about 130 children through the 12 hours I was working yesterday and it's it's an environment where how do you pinpoint who is actually stressed, who is going to be affected by trauma? (PID 007, Nurse Practitioner)

##### 3.1.2.1. Subtheme One: PTSD is not just reliant on injury severity

Clinicians highlighted that the psychological impact of an injury is not completely reliant on injury severity/mechanism. Some clinicians highlighted that families may be able to cope quite well after a severe injury, whilst some may experience emotional distress following a less severe injury.

Because you can get a child who has a small burn. That ends up at the parents could be on the ceiling, not coping, and you're giving quite a lot of support to those parents. Or you can have an uh, pedestrian versus car or like big poly-traumas, and the parents are quite chilled out. (PID 007, Nurse Practitioner)

Many clinicians emphasised that it is not possible to determine how children or families will react or be immediately aware of predisposing/risk factors for psychological distress upon patient presentation. They expressed that it would be unfair to restrict support to only children who have very severe injuries because emotional distress is a subjective and personal experience. Therefore, they advocated for offering a psychosocial intervention to all families of injured children.

And stress and like pain is very, very subjective. You know, and it's hard to say what isn't such a big deal to, to us, looking at them (.) you know, it could be quite a traumatic situation for an individual, um, families and it would be very, very hard to just assess that. (PID 012, Nurse Practitioner)

Clinicians also discussed that children and families may not display emotional distress within the ED and may experience the psychological impact when they have left the ED. Hence, it is difficult to determine the true extent of emotional impact when they are in the acute healthcare setting.

It's a challenge because it the, the (.) that depends on the individual impact to that person. You know that person could be there in front of you right now and, and might seem absolutely fine and completely. unphased by the situation. But actually it might have quite a significant impact on them in the days weeks months or years ahead (PID 022, Doctor)

##### 3.1.2.2. Subtheme Two: if the intervention is offered routinely then this could become part of the ED culture

Many clinicians noted that a universal approach to offering a psychosocial intervention could facilitate its integration into routine practice.

But I think if we're saying that this is something that might be considered and started in the emergency department and it should just be a blanket approach. The perk of that also is that you get staff that are really familiar with the process and then that is just part of the way they deliver care. Instead of kind of thinking, or does that person meet criteria? (PID 006, Nurse)

This was seen as a way to encourage a shift in providing mental health within the ED culture itself. Embedding psychological wellbeing into routine practices alongside physical care was viewed by some clinicians as a way to integrate mental healthcare support feasibly and sustainably without detracting from physical care.

Yeah, I mean, it's about embedding it in the organization and making it very routine and very much part of, of just the same way that you would do a set of observations (.) you know, you offered this resource for trauma informed care. (PID 022, Doctor)

#### 3.1.3. Theme Three: how to implement an intervention into an environment with limited capacity

Clinicians highlighted that capacity was a significant barrier to the implementation of a psychosocial intervention. Namely, time constraints and staff shortages were highlighted across interviews. These barriers were seen as significant threats to the feasible and sustainable implementation of an intervention. Furthermore, some clinicians felt that implementing an intervention may not be appropriate, given the difficulty already being experienced in providing medical care.

I'm paediatric researcher. So, one of the things that I'm very aware of, any departments right, I go to any of the departments is trying my best to not put additional burden on the nursing staff because I'm already aware they're chronically understaffed. (PID 003, Nurse)

However, clinicians were open to discussing ways in which a psychosocial intervention could be delivered feasibly, sustainably, and appropriately.

##### 3.1.3.1. Subtheme One: the intervention needs to be brief and accessible

In light of these barriers, clinicians discussed ways to provide psychosocial support in a way which is acceptable and feasible. Many clinicians expressed the importance of psychosocial support being provided in the form a brief and accessible intervention/resource, and something which could be easily and quickly offered to families and children.

Emergency Department is a busy place, but having said that, when patients and you know, children and families come to the emergency department with an issue and they're very receptive, so it would be a good place to start things because they might then consider it … I think it will be just it'll be a good thing to prime them, give them some ideas and give them some information leaflets. (PID 004, Doctor)

Clinicians reinstated the need to prioritise time for medical needs over mental health needs in the acute healthcare setting. Therefore, a brief intervention focusing on signposting and providing simple information for families to take away was proposed as a potential idea.

If it was a something that was small and didn't take a lot of time that they could then signposted to that, that could be provided. I think providing actual mental health support in a trauma situation probably wouldn't be able to be done, you know, like I think, you know, long taken along cause obviously that's something that takes time and requires focus and that's not either of things which we have in any. (PID 008, Doctor)

Across interviews, clinicians viewed electronic sources, including phones or websites, as a solution to creating something which was streamlined and accessible for both clinicians and families. An electronic medium was viewed to not be as time-consuming as face-to-face support, and therefore, would not detract from medical procedures.

I think if there was a website an app or some physical thing that we could give them that has that information on, then that might be a totally appropriate … If it was a something that was small and didn't take a lot of time that they could then signposted to that, that could be provided. (PID 008, Doctor)

Clinicians also highlighted that many families have access to smartphones and therefore, an electronic resource could be easily accessible. Some emphasised that families may not have the emotional capacity or time to access something within the ED, whereas something on their smartphone could be accessed in their own time.

I think the best mode of delivery is via an app. Most of the parents of children are very used to mobile phones and apps and that's the way they live their lives and communicate. (PID 016, Nurse)

Clinicians also viewed electronic resources as being more effective in terms of accessibility and uptake compared to printed resources. Printed resources such as leaflets, on the other hand, were repeatedly highlighted as ‘getting lost’ or forgotten.

But one of the things that we often do is at discharge, here's a leaflet to tell you more information about the condition, what brought you to hospital, what we've done in emergency since it's look out for going forward. Umm. They're given out. They're not necessarily taken out of the department. And again, that's, you know, parents are busy. They're getting all their stuff back together again and they're moving on. And some of them will have read it, hopefully absorbed it and left it there. Other ones may not have and may have missed it on leaving the department. (PID 003, Nurse)

The majority of clinicians encouraged the use of QR codes. QR codes were viewed as a useful tool to making information more easily accessible. Interviewees shared positive experiences of using QR codes when providing patients and families with medical information, and expressed the ease with which patients can access information.

I think it is the right environment. I just think like as you say the limitation is capacity. And so my thoughts would be that you kind of moving away from leaflets and would be to have something like a QR code is very popular at the moment and I guess the great cause you can have them laminated on a card in your pocket, but it's something where the family can access via QR code or a website and the details to like a video that could be accessed in different languages. (PID 009, Doctor)

##### 3.1.3.2. Subtheme Two: the intervention must be delivered after pain management

Clinicians also considered the role of capacity when identifying an appropriate time to offer an intervention. They highlighted that both clinicians and parents have limited capacity when a child arrives at the ED as the priority is physical medical stabilisation and recovery. Therefore, there is little capacity to offer a psychological intervention early on in the visit.

You would hope that you have this pain under control because if your child is in significant pain then we would give them nasal diamorphine or fentanyl or ketamine or something or inhaled nitrous oxide to help with that pain. And you would hope to make them comfortable during that first consultation that will be the aim you know, so as an immediately as that bit is kind of addressed as part of the rest of the care that child then I think it would be reasonable to provide the information. (PID 008, Doctor)

Clinicians expressed that parents will also have limited emotional capacity around this. Therefore, offering something initially – especially within an acute setting ED which can be busy and overwhelming – would not be feasible.

And what's really difficult in that scenario is trying to upskill the parent or caregiver in a timely fashion such that they're able to do it within the emergency departments, like there's two issues, isn't there? There's the ability to do this immediately. But also recognizing that they may not be able to do it immediately, but we can educate them to do it going forward. (PID 002, Doctor)

Therefore, clinicians expressed that a psychological intervention should be introduced after the necessary medical procedures have been completed. Some clinicians suggested that there is greater capacity around patient discharge as they already provide parents with advice regarding their child’s physical recovery around discharge and therefore, providing psychological advice alongside this could be feasible.

I'd be giving them information about how to do a dressing or or I'd be giving them information about how to use crutches, what, why not, how to give them advice about how to deal with the child going forward if it's that easy. (PID 001, Doctor)

Some clinicians also noted that parents may have more emotional capacity once their child is physically stable. Therefore, they may be more receptive to receiving information around discharge.

So I would probably offer it when stress levels can be deescalated as much as possible because. Nine times out of 10, when someone stressed they don't digest what information you're giving them anyway, so. Giving them that information that they can take away and upload when they're ready in the house. (PID 007, Nurse Practitioner)

However, some expressed that the timing of when to offer the intervention depends on the complexity of each case, as well as capacity within each hospital. Certain cases may be more complex and therefore, more time may be needed for certain medical procedures, or a child may need to be transferred to a different ward or department. Therefore, it may not be possible to identify a specific time to offer the intervention.

It depends on how injured the child is to a certain degree, certain interventions are going to take precedence and they're going to be prioritized according to what the medical staff see fit. (PID 002, Doctor)

#### 3.1.4. Theme Four: team-centred intervention training and delivery

Many clinicians highlighted that not one role/profession should solely be trained in intervention delivery and, instead, they emphasised the value of adopting a team-centred approach to training and implementation. This was seen as important as it would enable all staff to have the ability to provide psychosocial support and share the workload, and therefore, easily embed a psychosocial intervention into practice. In line with this, clinicians expressed the importance of good education and training for clinicians to ensure uptake of an intervention, and for improving clinician confidence. Clinicians felt that all staff should be trained to delivery an intervention in order to ensure adequate uptake and facilitate integration into routine care.

##### 3.1.4.1. Subtheme One: Not all staff are permanent

A key reason for adopting a team-delivered approach was because not all clinical staff are permanent – particularly doctors who are likely to have temporary placements. Therefore, some clinicians suggested that staff across different professions should be able to deliver an intervention.

So, I think unfortunately the doctor's coming through, they rotate quite quickly through the emergency department … So, I would like to see a combination of all teams really. (PID 016, Nurse)

A number of clinicians also identified play therapists as being ideally placed as their role already includes helping children and families through difficult emotions. However, it was also highlighted that not all EDs will have play therapists or may not in the ED every day. Therefore, it would not be appropriate to only train play therapists.

I don't think that um I don't think that it's helpful to and limit it to one um particular role. Well, because I think often the nurses staff build more of a rapport with the family because they spend more time with them, play therapists and sometimes involved, but are not always present. (PID 009, Doctor)

##### 3.1.4.2. Subtheme Two: nurses are well placed to offer the intervention but have a reduced capacity

The majority of clinicians identified nurses as having the right skillset to identify individuals in distress. Specific skills highlighted across interviews included nurses’ use of positive language and positive reinforcement, and simply having a greater level of contact with families and patients compared to doctors.

I think anybody who engages with parents and can have that rapport with them I think will be OK to refer because … a consultant might not spend as much time as a nurse would have on the board with the parents and stuff. (PID 004, Doctor)

However, many clinicians highlighted that nursing teams experience significant pressures and may not have capacity to provide an extra level of care.

But then we know the pressures on the nursing workforce and the establishment of the nursing workforce. We've already got a workforce that can't succinctly cover the care needs of the patient work that we're seeing. And it's just how we manage that, and managing new things with people to deliver. (PID 013, Nurse)

Therefore, many clinicians suggested that all members of the clinical team should be able to offer the intervention, and that the pressure to deliver a psychosocial intervention should not only be placed on nurses.

I think whoever has built a rapport with the patient or parents would be the best. That’s often nurses but sometimes they’ll come in and see a doctor straight away and it will all be like medical team led or the nurse practitioners will do everything from start to finish. So, I think it would have to change on a case-by-case basis, but whoever has built a rapport with the family. (PID 010, Nurse)

##### 3.1.4.3. Subtheme Three: all clinicians should be trained

Many clinicians suggested that training all clinical staff to deliver an intervention would be an effective way to embed it into existing care practices and become part of the ED culture. Therefore, the pressure does not fall on a single profession or member. Hence, psychosocial support can be delivered according to whoever builds rapport with the family which can be dependent on time constraints and case complexity.

I suppose the biggest barrier would be in any ED is the pressures of the department at the time (.) and I think that again, that comes back to the importance of embedding it in the culture, so that you're not relying on one person or another to provide the intervention any one time because it will literally be whoever just happens to have that, that 5 minutes of that time to support that family (.) (PID 023, Doctor)

The majority of clinicians highlighted the value of informative education on a psychosocial intervention. Key areas which they thought would need to be covered included the rationale behind an intervention the effectiveness of existing, similar interventions, and examples of what the families would be receiving.

As part of that training, it's ensuring that training gives them the reason to give to families (.) as to why it's working, that makes sense, and what the rationale is, and I think it's got to be training at a level that encompasses a lot of different kind of people. It's targeting both kind of our juniors in workforce and our senior nursing workforce and making sure that everyone understands and appreciates why (.) and everyone's got the skill set to do that. (PID 013, Nurse)

## 4. Discussion

The purpose of this study was to explore ED clinicians’ views on the feasibility, acceptability, and practicalities of implementing a psychosocial intervention for children and families following acute paediatric injury. The interviews and focus groups revealed that clinicians recognised the need to support patients and their families following acute injury, but that a potential intervention must consider the pressures present within EDs. A solution to working around these barriers was to design a brief and accessible intervention which could be offered to all families of injured children by all clinicians across different professions. Critically, ED clinicians felt that effective implementation would involve embedding an intervention into the ED culture and routine practices.

Whilst participants highlighted that pressures within the ED make it difficult to provide psychosocial support, they acknowledged their unique position in being able to provide early psychological care. The importance of emotional and psychological support from medical teams is highlighted in models conceptualising PTSS following acute paediatric injuries (Kazak et al., 2006; Marsac et al., 2014). Indeed, research suggests that early cognitive appraisals, avoidant coping, and support from parents and the community (e.g. medical clinicians) during this peri-trauma period can influence children’s development of PTSS (Ehlers et al., 2003; Trickey et al., 2012). Thus, whilst there are barriers to delivering psychological support in the ED, there is value in developing a feasible and sustainable intervention which mobilises opportunities for clinicians to psychologically support children and families. Types of support can range from screening and assessment, to direct care and intervention (Kazak et al., 2006; Marsac et al., 2016).

However, clinicians emphasised that effective implementation of routine psychosocial support would involve embedding a potential intervention into the ED culture. This was seen as a potential solution to providing psychosocial support without placing additional burden upon clinicians in the ED. This aligns with research which suggests that factors relating to capacity, including a lack of dedicated space and resources, and time constraints, contribute to clinicians’ perceptions of their ability to provide psychosocial support to injured children and their families (Afzal et al., 2022; Alisic et al., 2016; Kassam-Adams et al., 2015). Therefore, clinicians advocated for embedding the intervention into existing ED practices and the culture. This aligns with the ‘trauma-informed’ approach to paediatric healthcare (Marsac et al., 2016). This is defined as embedding a basic understanding of the impact of trauma and pathways to recovery into an organisation at all levels (Substance Abuse and Mental Health Services Administration, 2014). This approach involves four key principles: (1) realising the widespread influence of trauma and pathways of recovery; (2) recognising the signs and symptoms of trauma in individuals; (3) responding, as guided by knowledge of trauma which has been integrated into policies, procedures, and practices; and (4) actively resisting retraumatisation. The ‘D-E-F’ protocol provides a framework for incorporating a trauma-informed approach into paediatric healthcare systems following paediatric illness (Stuber et al., 2006). Here, physical health is initially addressed (the A-B-C’s) and then healthcare providers can move onto patients’ psychological wellbeing recovery by reducing Distress, providing Emotional support, and including the Family (the D-E-F) (Marsac et al., 2016; Stuber et al., 2006). Thus, adopting a trauma-informed approach to acute paediatric healthcare could enable the provision of psychosocial support following paediatric injury.

One pathway suggested by clinicians to embed psychosocial care into routine ED practices involves a blanket approach to implementation. This applies to both training all members of staff and offering this support to all families of injured children. This aligns with the ‘universal trauma precautions’ approach, which involves providing training to all medical staff to ensure a multi-agency awareness, recognition, and prevention of psychological trauma (Raja et al., 2015). Recommendations include implementing basic principles of psychological trauma and trauma-informed care into training to ensure that all staff are aware of psychological trauma presentations and are equipped to provide basic support (Bruce et al., 2018; Butler et al., 2011). Once all staff are equipped with an understanding of psychological trauma and its widespread impact, this can then create an opportunity to embed this awareness into the culture.

Participants also had specific recommendations regarding training for clinicians to deliver a novel intervention for paediatric PTSS. Across interviews, clinicians expressed that training should outline the rationale behind the intervention. This aligns with recommendations that education should include the magnitude of trauma in patients, the impact of trauma and how to effectively utilise trauma-informed practices (Marsac et al., 2016). The importance of effective education and training when introducing novel interventions has been highlighted as a way to engage staff, and establish support, confidence, and self-efficacy which, in turn, will encourage a more effective and sustainable implementation of novel interventions into hospitals (Damschroder et al., 2009; Geerligs et al., 2018). Providing such training for all clinicians is important as many healthcare staff are not confident in their knowledge and skills in providing care to distressed child and their families (Afzal et al., 2022; Alisic et al., 2016; Simons et al., 2022), and this lack of knowledge can be a significant barrier to the implementation of interventions into hospitals (Geerligs et al., 2018). Therefore, findings from the current study and wider literature provide a strong case for future research to also develop accessible and universal training for medical staff, alongside core intervention development.

As well as a universal approach to clinician training, the majority of clinicians believed that all families should be offered the opportunity to access a psychosocial intervention. As highlighted by many clinicians in the present study, psychological distress in children and families is not always related to the objective injury severity (Brosbe et al., 2011; Kazak et al., 2006) and clinicians’ judgement of which children may be vulnerable to experiencing psychological distress may not be accurate (Brown et al., 2022). This perspective further aligns with the universal precautions approach which highlights that trauma-related difficulties can be present in all patients (Raja et al., 2015; Substance Abuse and Mental Health Services Administration, 2014). Hence, an intervention should be designed in a way that it can be easily offered by all clinicians to all families of injured children.

The consideration of intervention format and design was highlighted by clinicians as a crucial component of successful implementation. Research suggests that trauma-informed interventions for paediatric healthcare must be designed to be easily integrated into existing models of care (Geerligs et al., 2018; Kazak et al., 2006; Stuber et al., 2006). In particular, clinicians felt that any intervention would need to be brief and accessible. Clinicians specifically recommended the value of developing an electronic intervention which can easily be offered by clinicians and accessed by families via QR codes. Wider literature proposes that digital mental health services are an accessible and cost-effective way to providing mental health support to children and young people (Hollis et al., 2017; Lyon et al., 2020; Naslund et al., 2019). Specific recommendations by clinicians involved developing an app or website which they could offer to families alongside medical advice. This was also seen as a way to reduce pressure on families as they could access it in their own time, potentially following acute medical stabilisation or hospitalisation. Therefore, future research can aim to adapt existing educational interventions for paediatric PTS (e.g. (De Young et al., 2016) for electronic devices. This can provide the opportunity to integrate accessible and streamlined psychosocial care into UK and Irish EDs which does not place added pressure on clinicians and families.

However, limitations of the present sample must be considered. Firstly, the sample is relatively small with a total of 24 participants recruited from a paediatric emergency research network. This may limit the representativeness of the sample as the participants who volunteered may already have favourable views of providing psychosocial support in EDs. Secondly, the majority of clinicians were from the UK and were either doctors or nurses. This means that perspectives of Irish clinicians and clinicians from other ED professions, such as allied health professional (e.g. occupational therapists and play therapists), mental health care emergency teams, and technicians were under-represented. This presents a particular limitation as a universal precautions approach requires training all staff in being able to offer trauma-informed care. Future research can benefit from recruiting clinicians from a larger pool of sites across the UK and Ireland and across diverse clinical roles to obtain representative perspectives. This can inform future intervention development and implementation strategies.

Furthermore, whilst the present investigation provided valuable insights into ED clinicians’ perspectives on providing paediatric psychosocial care following acute injury, future research must seek to bridge the gap between theory and implementation. Specifically, future research can aim to develop implementation strategies for a psychosocial intervention. Potential avenues for future research include adapting existing interventions (e.g. De Young et al., 2016; Kassam-Adams et al., 2016) for a UK and Irish healthcare setting, developing accessible training material for clinicians, and conducting in-depth interviews with a larger, more representative pool of clinicians to inform implementation strategies. This work can form a critical step in implementing trauma-informed care into paediatric EDs.

To summarise, our findings provide insight into UK and Irish ED clinicians’ perspectives on implementing psychosocial support for acutely injured children and their families. The interviews and focus groups revealed four key themes which are consistent with trauma-informed care and the universal precautions approach to trauma-informed care. The majority of clinicians advocated for an accessible intervention which can be easily implemented in existing healthcare practices, and something which all clinicians can be trained to deliver to all families This information can support future research in developing a digital intervention for paediatric PTSS, as well as further qualitative work with a larger, more representative pool of ED clinicians to inform an evidence-based implementation strategy.

## Supplementary Material

Supplementary_Material_A.docx

## Data Availability

The authors confirm that the data supporting the findings of this study are available within the article and its supplementary materials.
