# Supplementary material for: Emergency department clinicians’ views on implementing psychosocial care following acute paediatric injury: a qualitative study
Source: Eur J Psychotraumatol. 2024 Jan 10;15(1):2300586. doi: 10.1080/20008066.2023.2300586 (PMC10783840; doi:10.1080/20008066.2023.2300586)
Supplement: Supplementary_Material_A.docx [file ZEPT_A_2300586_SM0358.docx]

| **Supplementary Material A**  Themes and Sub-Themes and Sample Quotations | |
| --- | --- |
| **Theme One: Mental health support for families is needed in the ED** | |
| Subtheme: EDs can also consider the emotional impact of injury. | We're giving anaesthesia, we’re monitoring patients, but we’re monitoring multiple patients and you know managing them all as best we can. So, in the times that we are not at the patient’s side, giving parents something that is going to educate them on the impact of trauma and what they can do to better understand those impacts and how they can, um, not change their behaviour, but how they can manage that as best as possible and I think it's really, really valuable. (PID 003, Nurse)  I think it is an area where we probably fail to and fully meet children and families’ needs. I think we see children and they've been involved in quite high mechanism accidents. And we often do their scan and their scan comes back negative and we feel quite relieved that we can kind of turn them around and get them home often and probably don't think a huge amount about what the aftermath from a psychological perspective is and yeah, I think it probably is more important than we give it credit, really. (PID 009, Doctor) |
| Subtheme: Opportunities for early intervention in the ED | I think it's having that early opportunity to, to intervene and support at that (.) umm, point that that that young person is either, you know, at their most vulnerable or with the most questions, and the most in crisis, most in need of that support. And so, you jumping in at that very early opportunity is gonna make all the difference, I think, to being able to support that child, and their family going forward. (PID 023, Doctor)  I think it sounds like the emergency department is the ideal place to to be the catch all location for the conditions that you're trying to, I suppose empower parents and inform parents how to manage their child's symptoms. (PID 002, Doctor)  And I think probably you know if you're looking at supporting families to provide emotional support for a child that's been involved in a trauma, probably the time that you're going to get parents attention around that is probably in the emergency department. And so I think it is the right environment. (PID 009, Doctor)  And so there’s a place for kind of going, “You know there’s this thing out here and here’s some signposting to it,” but recognizing that maybe they’re not going to engage with it right now because they’re too busy processing the fact that this isn’t the way they expected their day to turn out. (PID 018, Doctor) |
| Subtheme: Parents need support | And sometimes I think also the scenario or the mechanism that led to the injury, I've seen a lot of times and if parents have had some sort of a contributing factor or even if they haven't, they often tend to blame themselves for not being around or for, you know, anything that they could have done preventative to have you avoided them from being in that situation. In those situations I think it's kind of important to be able to give even parents some support. It's not just a child, I think. Parents do have a lot of impact, uh themselves. (PID 005, Physiotherapist)  So once these patients have left hospital, we ring them up and just see how they're getting on and more and more. It's it's parents that it's later down the line or actually still having flashbacks about this. They're struggling with that and it's parents often contact us to ask exactly that. How can I support my child. (PID 004, Doctor)  But what I think I do recognize is that parents, particularly when they've something significant has happened, can feel quite helpless. …. When you give parents quite robust information, I think they go home feeling more comfortable and I imagine reducing parental anxiety is likely to have a ripple effect throughout the household and also to the child. So I imagine there's a probably a huge benefit and the kind of family attitude towards the event I imagine. (PID 009, Doctor)  And you know these kids and families just goes through a lot. And it's often when we do follow up phone calls to children and their families when they're discharged after two weeks that they've been discharged from hospital and these are like quite significant injuries, major trauma injuries and a lot of the families do struggle and they say the care has been great. But actually when they get home, they do struggle. And I think that would be really, really useful. And I think it would be nice to get sort of availability for us as trauma coordinators to have that facility as well. (PID 011, Nurse) |
| **Theme two: The benefits of a universal approach to implementation** | |
| But sometimes it's just not right for that particular young person or child at that time, and as long as you can identify that and say OK here are the resources, you don't need to access them now, don't worry about it, but take this away with you (.) so at some point, if you want to, then you can access these resources. Because by that time, the (.) the face-to-face contact with me or the other clinician or, or whoever it is has been lost. You're not gonna get that a year later or six months later, but at least you've got the resources. (PID 024, Nurse)  It's difficult really because stress will kind of affect people so differently. It's kind of who do you target to give the advice to. [The] ED is ridiculously busy. I think we saw about 130 children through the 12 hours I was working yesterday and it's it's an environment where how do you pinpoint who is actually stressed, who is going to be affected by trauma? (PID 007, Nurse Practitioner | |
| Subtheme: PTSD is not just reliant on injury | Because you can get a child who has a small burn. That ends up at the parents could be on the ceiling, not coping, and you're giving quite a lot of support to those parents. Or you can have an uh, pedestrian versus car or like big poly-traumas, and the parents are quite chilled out. (PID 007, Nurse Practitioner)  Speaker 1: And stress and like pain is very, very subjective. You know, and it's hard to say what isn't such a big deal to, to us, looking at them (.) you know, it could be quite a traumatic situation for an individual, um, families and it would be very, very hard to just assess that. (PID 012, Nurse Practitioner)  I would say everybody. I worked on a burns unit for 3 years before working in A&E and sometimes the most distressed parents were the ones who had the tiniest injuries and who were going to be discharged the next day. So yeah, I think that you never know how a parent is going to react in the injury and following the injury, especially if it’s like something that they could have prevented which most accidents are. (PID 010, Nurse)  You know, there are some kids and families that will have had a really scary, horrible experience, but have actually come out of it physically quite ok and may well be going home from us and yeah then I think we’re the ideal place, because we’re the only place they may have contact with for you know a week or so, it depends on the local fracture clinic or whatever (PID 017, Doctor)  I think I think you're probably better having a system where you can offer it to everyone or at least screen everyone. Because there's so many other factors, I think like we've got a lot of... Within trauma especially, we find that children that are predisposed to trauma are possibly also predisposed to struggling with mental health because they're from lower socioeconomic areas. A lot of them have got ADHD or ASD and then they're kind of prone to risk taking behaviour. A lot of them are already known to CAMHS. And so I think even some of the ones that you maybe the mechanism to us, wouldn't seem particularly traumatic it, you know, you don't know how it's gonna impact on their point in life and what might already be going on at school and and it might just make things a lot worse. So I do think there's not a simple way to say oh "you've had this type of trauma so you're gonna need it". So I think probably looking at screening everyone or offering it to everyone. (PID 005, Physiotherapist)  It's a challenge because it the, the (.) that depends on the individual impact to that person. You know that person could be there in front of you right now and, and might seem absolutely fine and completely. unphased by the situation. But actually it might have quite a significant impact on them in the days weeks months or years ahead (PID 022, Doctor)  …Even if it's a minor trauma, you might find that a child reacts more significantly to it than a child who's had a severe trauma, who hasn't reacted to it. So I think if youre intervention is based is aimed to alleviate that post traumatic stress episode associated with the trauma, then it makes sense to offer it to anybody who you think would come in the category that might be so affected. (PID 008, Doctor) |
| Subtheme: If an intervention is offered routinely then this could become part of the ED culture | But I think if we're saying that this is something that might be considered and started in the emergency department and it should just be a blanket approach. The perk of that also is that you get staff that are really familiar with the process and then that is just part of the way they deliver care. Instead of kind of thinking, or does that person meet criteria? (PID 006, Nurse)  Yeah, I mean, it's about embedding it in the organization and making it very routine and very much part of, of just the same way that you would do a set of observations (.) you know, you offered this resource for trauma informed care. (PID 022, Doctor)  I suppose the biggest barrier would be in any ED is the pressures of the department at the time (.) and I think that again, that comes back to the importance of embedding it in the culture, so that you're not relying on one person or another to provide the intervention any one time because it will literally be whoever just happens to have that, that 5 minutes of that time to support that family. (PID 023, Doctor)  We have trauma paperwork for all children that come through the department, so it could be on, you know, the front page that offer this to parents because there's a nursing booklet. So, I think definitely A&E is the place and....That designed well, it could be something that doesn't need to take any time. (PID 009, Doctor) |
| **Theme three: How to implement an intervention into an environment with limited capacity** | |
| I'm paediatric research. So, one of the things that I'm very aware of, any departments right, I go to any of the departments is trying my best to not put additional burden on the nursing staff because I'm already aware they're chronically understaffed. (PID 003, Nurse) | |
| Subtheme: The intervention needs to be brief and accessible | Emergency Department is a busy place, but having said that, when patients and you know, children and families come to the emergency department with an issue and they're very receptive, so it would be a good place to start things because they might then consider it, but you might not be able to get through the whole process in emergency department. I think it will be just it'll be a good thing to prime them, give them some ideas and give them some information leaflets. And I think because they're very receptive in that period, I think it will have a good impact. (PID 004, Doctor)  To get the answers that you need, to elicit the information you need, and it's just (.) I think we're gonna start adding that to clinical assessment and clinical kind of workload (.) it's finding a succinct way to ask the questions. (PID 013, Nurse)  If it was a something that was small and didn't take a lot of time that they could then signposted to that, that could be provided. I think providing actual mental health support in a trauma situation probably wouldn't be able to be done, you know, like I think, you know, long taken along cause obviously that's something that takes time and requires focus and that's not either of things which we have in any. (PID 008, Doctor)  I think probably parents will be happy to know it's there, but I think at that stage that's not what they are focused on. So I think you know, knowing that it's available and it's kind of it's coming, but I think. That's all they might build to take in at that point, and and a leaflet or something, you know, just as a reminder that. That's something that will kind of be ongoing is probably a good thing. (PID 005, Physiotherapist)  I think if there was a website an app or some physical thing that we could give them that has that information on, then that might be a totally appropriate... If it was a something that was small and didn't take a lot of time that they could then signposted to that, that could be provided. (PID 008, Doctor)  I think the best mode of delivery is via an app. Most of the parents of children are very used to mobile phones and apps and that's the way they live their lives and communicate. (PID 016, Nurse)  But one of the things that we often do is at discharge, here's a leaflet to tell you more information about the condition, what brought you to hospital, what we've done in emergency since it's look out for going forward. Umm. They're given out. They're not necessarily taken out of the department. And again, that's, you know, parents are busy. They're getting all their stuff back together again and they're moving on. And some of them will have read it, hopefully absorbed it and left it there. Other ones may not have and may have missed it on leaving the department. (PID 003, Nurse)  I think it is the right environment. I just think like as you say the limitation is capacity. And so my thoughts would be that you kind of moving away from leaflets and would be to have something like a QR code is very popular at the moment and I guess the great cause you can have them laminated on a card in your pocket, but it's something where the family can access via QR code or a website and the details to like a video that could be accessed in different languages. (PID 009, Doctor)  So I think if it could be electronic, that would be great. I assume there's and also that means it's more accessible widely spread opposed to saying or it's a face to face service. Yes, too much. Think about is probably unrealistic. How do you cater for a larger region? And I think something that is electronic is then transferable across trusts, cross board and also means that if it the ball is started in one center and then move to another, then the messaging is the same it's access the same way. (PID 006, Nurse) |
| Subtheme: The intervention must delivered after pain management | Well, I mean, I mean you were, you would hope that you have this pain under control because if your child is in significant pain then we would give them nasal diamorphine or fentanyl or ketamine or something or inhaled nitrous oxide to help with that pain. And you would hope to make them comfortable during that first consultation that will be the aim you know, so as an immediately as that bit is kind of addressed as part of the rest of the care that child then I think it would be reasonable to provide the information. (PID 008, Doctor)  And what's really difficult in that scenario is trying to upskill the parent or caregiver in a timely fashion such that they're able to do it within the emergency departments, like there's two issues, isn't there? There's the ability to do this immediately. But also recognizing that they may not be able to do it immediately, but we can educate them to do it going forward. (PID 002, Doctor)  I guess there is a balance there isn't because you don't want to be. If it depends on how injured the child is to a certain degree, certain interventions are going to take precedence and they're going to be prioritized according to what the medical staff see fit. But there is a stage that you get to you in pain is being addressed and people are more settled in that you are able to introduce tools like this to to help the parents. (PID 002, Doctor)  I'd be giving them information about how to do a dressing or or I'd be giving them information about how to use crutches, what, why not, how to give them advice about how to deal with the child going forward if it's that easy. (PID 001, Doctor)  However, if the injury is so severe where the child for example, attended, requiring resuscitation level care or care that was incredibly intense. Whether or not the timing of that would be more appropriate once the family are settled and that child is in a more safe environment- not that the emergency department isn't safe, but with regards to the fact that they're injury is may be cared for, what has had its immediate intervention, whether the family would then be more receptive of help. (PID 006, Nurse)  When everything that you know when all the whole things calmed down and just before discharge, I would say to the parent "you've had quite a traumatic experience today, the both of you, please will you scan this QR code? And even if you want to take it home, give it a good read. So when you're when you're less stressed, you're out of the environment, you can think you can digest." So I would probably offer it when stress levels can be deescalated as much as possible because. Nine times out of 10, when someone stressed they don't digest what information you're giving them anyway, so. Giving them that information that they can take away and upload when they're ready in the house. (PID 007, Nurse Practitioner)  It depends on how injured the child is to a certain degree, certain interventions are going to take precedence and they're going to be prioritized according to what the medical staff see fit. (PID 002, Doctor)  I think the families would and the child needs to have the physical care primarily, but again, you know the psychological care of the child should run alongside that as well. But I think if you were asking a family to do that before they've had their physical care, that maybe wouldn't be appropriate… I think after they’ve had the diagnosis and the treatment is commenced or nearly finished, that would be a good time. (PID 015, Psychologist)  think when once the acute situation has been dealt with and I think parents know that they're not in a life threatening condition or, you know any other situation, which will probably take up most of their um thought process and stuff. (PID 004, Doctor) |
| **Theme four: Team-centred intervention training and delivery** | |
| Subtheme: Not all staff are permanent | So, I think unfortunately the doctor's coming through, they rotate quite quickly through the emergency department. So, would maybe forget to provide that information because they're not as familiar as the permanent members of staff, if they've you know, just been there a couple of weeks, they're not going to know that that is available. So, I would like to see a combination of all teams really. (PID 016, Nurse)  I don't think that um I don't think that it's helpful to and limit it to one um particular role. Well, because I think often the nurses staff build more of a rapport with the family because they spend more time with them, play therapists and sometimes involved, but are not always present. (PID 009, Doctor)  You know, the docs we rotate in and out on a shift basis, and even the people that we have to rotate in and out change every four months. So although we can make them aware and the longer term staff will get the hang of it, the group that will really be the bang for your buck is the kids ED nurses. (PID 017, Doctor) |
| Subtheme: Nurses are well placed to offer the intervention but have a reduced capacity | I think anybody who engages with parents and can have that rapport with them I think will be OK to refer because... a consultant might not spend as much time as a nurse would have on the board with the parents and stuff. (PID 004, Doctor)  But then we know the pressures on the nursing workforce and the establishment of the nursing workforce. We've already got a workforce that can't, can't succinctly cover the care needs of the patient work that we're seeing. And it's just how we manage that, and managing new things with people to deliver. (PID 013, Nurse)  I think whoever has built a rapport with the patient or parents would be the best. That’s often nurses but sometimes they’ll come in and see a doctor straight away and it will all be like medical team led or the nurse practitioners will do everything from start to finish. So, I think it would have to change on a case-by-case basis, but whoever has built a rapport with the family. (PID 010, Nurse)  Of course, our triage nursing staff, who can immediately recognize a child that perhaps meets the criteria and as part of the finishing off of their triage process they'll address whatever needs that need to be addressed, such as pain or immediate interventions, but may also show the parents this resource. And perhaps under smartphone, which would be a really good way of doing it. And say listen, have a read of this while you're waiting for the next stage in your care (PID 002, Doctor)  We obviously us nurses, we triage them at the start. We do all the dressings we do (.) So sometimes we've got a better relationship with them than the doctors. (PID 011, Nurse)  And I would say it's probably gonna be placed with the nurses... I think it would probably be best placed with them and they're more of a permanent fixture and they've already got kind of the background of the paediatric tools and the more experience there. (PID 013, Nurse)  So I think like everyone says, the doctors move on quickly, don't they? … But then we know the pressures on the nursing workforce and the establishment of the nursing workforce. We've already got a workforce that can't, can't succinctly cover the care needs of the patient work that we're seeing. And it's just how we manage that and managing new things. (PID 014, Nurse) |
| Subtheme: All clinicians should be trained | So this is very difficult because of the clinical pressures, especially in the winter months. I think it should be a collaboration between the whole team. It shouldn't be one specific role for either medical or nursing staff. (PID 014, Nurse)  I don't think that um I don't think that it's helpful to and limit it to one um particular role. Well, because I think often the nurses staff build more of a rapport with the family because they spend more time with them, play therapists and sometimes involved, but are not always present. So, you know, one pay therapist in our department who who isn't there full time. And so I think probably it's everyone's responsibility and it should probably be done as part of the discharge planning for patients that have ended with traumatic injuries. (PID 010, Nurse)  It's, it's, yeah, about having it as part of the culture of your approach to towards those, those individuals and you know every member of the team being able to provide that support. And it's not about (.) my initial thought was you know if too many people are making that approach, is, is there a risk of bombarding that child and that family with that information, but actually, if you (.) if, if the training's in place and the culture's there and you understand why you're making, umm, that approach, then actually, a much more of a soft genuine introduction to the, to that service would work very well and yeah, and I don't think there's any one person. (PID 017, Doctor)  I think if I had a real good understanding of the (.) the intervention and I understood how it benefited the children and the families and that would certainly give me the confidence to intervene to support those children and families at any time (.) I suppose the biggest barrier would be in any ED is the pressures of the department at the time (.) and I think that again, that comes back to the importance of embedding it in the culture, so that you're not relying on one person or another to provide the intervention any one time because it will literally be whoever just happens to have that, that 5 minutes of that time to support that family (.) (PID 023, Doctor)  As part of that training, it's ensuring that training gives them the reason to give to families (.) as to why it's working, that makes sense, and what the rationale is, and I think it's got to be training at a level that encompasses a lot of different kind of people. It's targeting both kind of our juniors in workforce and our senior nursing workforce and making sure that everyone understands and appreciates why (.) and everyone's got the skill set to do that. (PID 013, Nurse)  I think it needs to be relatively simple to deliver, relatively quick to deliver, and also with that good education before we, you know, (.) as the department, if staff are educated and informed, we can embed stuff actually quite quickly and quite easily. We've shown that with recently with several projects. (PID 013, Nurse) |
